# An Overview of the Versatility of the Parts of the Globe Artichoke (*Cynara scolymus* L.), Its By-Products and Dietary Supplements

**DOI:** 10.3390/nu16050599

**Published:** 2024-02-22

**Authors:** Beata Olas

**Affiliations:** Department of General Biochemistry, Faculty of Biology and Environmental Protection, University of Lodz, Pomorska 141/143, 90-236 Lodz, Poland; beata.olas@biol.uni.lodz.pl; Tel./Fax: +48-42-6354485

**Keywords:** artichoke, *C. scolymus*, *Cynara scolymus* L., cynara

## Abstract

*Cynara scolymus*, also known as the globe artichoke or artichoke, is grown as a food, mainly in the Mediterranean, Canary Islands, and Egypt, as well as in Asia and South America. It has also been associated with various health benefits and is used in plant-based dietary supplements and herbal infusions. Its edible parts, consisting of the head or capitula, flower, and leaves, have shown various biological activities, including anti-cancer, hepatoprotective and antimicrobial potential. The leaves are mainly used in infusions and extracts for their health-promoting properties, although all their edible parts may also be consumed as fresh, frozen, or canned foods. However, its primary health-promoting activity is associated with its antioxidant potential, which has been linked to its chemical composition, particularly its phenolic compounds (representing 96 mg of gallic acid equivalent per 100 g of raw plant material) and dietary fiber. The main phenolic compounds in the heads and leaves are caffeic acid derivatives, while the flavonoids luteolin and apigenin (both present as glucosides and rutinosides) have also been identified. In addition, heat-treated artichokes (i.e., boiled, steamed or fried), their extracts, and waste from artichoke processing also have antioxidant activity. The present paper reviews the current literature concerning the biological properties of different parts of *C. scolymus*, its by-products and dietary supplements, as well as their chemical content and toxicity. The literature was obtained by a search of PubMed/Medline, Google Scholar, Web of Knowledge, ScienceDirect, and Scopus, with extra papers being identified by manually reviewing the references.

## 1. Introduction

The artichoke (*Cynara*), a member of the *Asteraceae* family, is perhaps the oldest vegetable grown by humans. It exists as various varieties, including *Cynara scolymus* L., *Cynara altilis*, and *Cynara sylvestris*; their harvest begins between autumn and winter, i.e., for larger artichokes intended for the fresh food market, and ends by July, for the industrial transformation phase [1,2]. The botanical name is derived in part from the tradition of fertilizing the plant with ashes (Latin: *cineris*, *cinis*) and partly from the Greek *skolymos*, meaning “thistle”, from the spines found on the bracts that enclose the flower heads and form the edible portion of the plant [3].

The plant can reach a height of 1.2 to 1.3 m. It consists of a stem with leaves covered with inflorescence baskets resembling thistle flowers. Each artichoke variety has unique characteristics, making one better for steaming and another suitable for frying. The world leader in artichoke breeding is Italy, which produced 389,813 tons in 2018, representing 40% of global production. They are followed by Egypt, which produced 323,866 tons that year, Spain (208,463 tons), and France (47,190 tons) [4]. Early varieties such as Spinoso Sardo, Catanese, Romanesco, and Terom differ from each other in appearance, flower size and flavor [5].

*C. scolymus*, the globe artichoke or artichoke, is grown mainly in the Mediterranean, the Canary Islands, Egypt and the countries of Asia and South America. It is often used in plant-based dietary supplements and herbal infusions for its beneficial health benefits. It has been used as a medical plant, especially as a remedy for digestive problems, since the fourth century BCE, alone or in combination with other medicinal plants, such as *Curcuma longa*, *Achillea millefolium*, and *Gentiana lutea*. The first information on the therapeutic potential of the artichoke can be found in works by the ancient Egyptians, Greeks, and Romans [3,5,6,7].

The edible part of *C. scolymus* consists of the head of its inflorescence, after the removal of the outer hardened scales of the casing, the remaining fleshy ends of the scaly casings, the inflorescence itself and its base, and the short end of the stem. As only the flower base is edible in older buds, the edible portion typically constitutes only 30–40% of the total biomass, with the stems and leaves being discarded as waste or used as animal fodder or fertilizer [2,3]. Despite being perishable vegetables, artichokes can be stored for up to two weeks from the time of harvest [5,8].

The edible parts are exemplified by the tender inner bracts, consumed as fresh, frozen, or canned food, and the receptacle. Artichokes are typically cooked, fried or baked, and in Italy, the young flower heads of the artichoke are often covered with olive oil after cooking and stored. Artichokes can also be canned or used as additions to salads [5]. It is important to note that while artichokes do not cause allergic reactions in most consumers, four cases of artichoke allergy have been described, manifested as airway obstruction and swelling of the tongue [9].

Artichoke and its products have gained attention in scientific circles. Different in vivo and in vitro studies indicate that they may possess various biological activities, which may be due to their chemical content, particularly their phenolic compounds, inulin, fibers and minerals [3,10]. The present paper reviews the current literature concerning the biological properties of different parts of *C. scolymus*, its by-products, and dietary supplements, as well as their chemical content and their toxicity. This literature was obtained by a search of PubMed/Medline, Google Scholar electronic databases, Web of Knowledge, ScienceDirect and Scopus, with extra papers being identified by manually reviewing the references. I used these databases to search the keywords “artichoke”, “*Cynara scolymus*”, “*C. scolymus*”, “cynara”, and “bioactive compound” or a combination of these terms, to obtain relevant articles and summarize them. The search was restricted to English-language publications, including review papers, articles, case reports, and abstracts from congress proceedings. The last search was run on 14 February 2024.

## 2. Phytochemical Characteristics of *C. scolymus*

**Figure 1:**
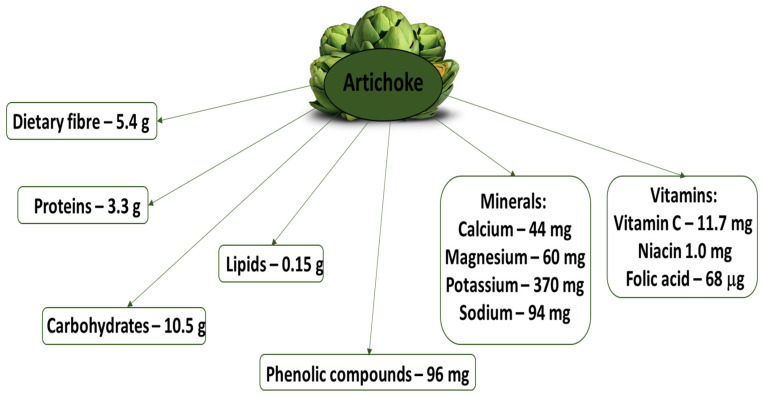
Nutritional value of 100 g of raw globe artichoke, based on Teterczyk and Michalak-Majewska [5].

The energy value for 100 g of raw artichoke is 57 kcal, which makes it a low-calorie vegetable. In addition, 100 g of raw artichoke also contains about 3 g of protein, 0.2 g of fatty acids and 11 g of carbohydrates, including as much as 5.4 g of dietary fiber (Figure 1). It is rich in water (91% of food portions) [5,11]. An important component of artichoke is inulin, which constitutes a major carbohydrate reserve in the storage organs (up to 36% dry matter); inulin is known to have a prebiotic effect [2,12], being found to stimulate the growth of *Bifidobacterium* strains. The dry matter of artichoke has also been found to consist of 6.7% sucrose, 3.2% glucose and 1.3% fructose, with the presence of frucotooligosaccharides (e.g., nystose and fructofuranosylpylystosis) representing 3.6 g/kg dry matter [5,8,11]. Artichokes are also a source of minerals such as calcium, magnesium, iron, zinc, potassium and vitamin C.

Artichokes are valued for the presence of numerous phenolic compounds, *viz.* chlorogenic acid, caffeic acid, ferulic acid, apigenin, luteolin and cynarin, representing 96 mg per 100 g of raw plant material, calculated as gallic acid equivalents. However, the main phenolic compounds in the heads and leaves are caffeic acid derivatives. The highest concentration of phenolic compounds is found in the flower base of the artichoke and the lowest in the bud [5,13,14]. In addition, these concentrations also differ between varieties [5,14], with the highest concentration of cynarin being noted in the outer leaves of the head of the Violetta di Sicilia cultivar (53.6 mg/kg dry matter) [15]. Moreover, under refrigerated conditions, the concentration of caffeic acid increases with storage time [5,11,14].

The fleshy flower base, the heart of the artichoke, contains tannins, which are responsible for the bitter taste, and anthocyanins such as cyanidin-3-glucoside, cyanidin-3,5-diglucoside and delphinidine glucoside, which determine its purple color [5,14]. The total concentration of anthocyanins in artichoke flowers is up to 1.7 g/kg dry matter [5,11,14].

Interestingly, artichoke by-products, including raw artichoke, thermally treated artichoke, i.e., blanched artichoke, and the waters from artichoke blanching, are also good sources of antioxidants, especially phenolic compounds [16]. For example, artichoke waste contains 24 g of phenolic compounds per 100 g dry weight (ethanolic extracts) and 15.4 g per 100 g of dry weight in aqueous extracts. Moreover, Llorach et al. [16] report the presence of 15.4 g of phenolic compounds per 100 g of dry weight in methanolic extract from raw artichoke and 9.9 g in aqueous extract, both expressed as caffeic acid derivatives. The authors also found 11.3 g of phenolic compounds per 100 mL of artichoke blanching water.

Phenolic compounds are also present in dried extracts of artichoke heads and artichoke pomace. For example, they are present in 12 g per kg of pomace dry matter [5,14].

Lim [14] reports a significant increase in the level of antioxidants, especially phenolic acids, in artichoke after boiling in water; however, no such significant effect was noted in steamed or fried artichokes. In addition, the amount of flavonoids in the artichokes decreased after heat treatment. The high water solubility of many artichoke phenolics may produce a lixiviation of these compounds, which will be extracted from the plant and be part of the boiling water.

The aroma of cooked artichoke is primarily due to the sesquiterpenes β-selinene (40%) and caryophyllene (19%). Interestingly, the hydroxyl triterpenes, fardiol and taraxasterol have been isolated from artichoke flowers [5,14].

It is important to note that the inedible parts of the artichoke (e.g., leaves or stems) are also abundant in biologically active compounds. For example, the leaves are a rich source of Si, Fe, Na, K, Ca, Cu, and Mg, inulin, ascorbic acid, folate, and various phenolic compounds. Artichoke leaves are the richest source of luteolin of all its parts. Moreover, the concentration of the phenolic compound cynarin increases as the plant grows (from 0.01 to 0.05% dry matter) [5,14].

Lim [14] reports that the stems and leaves of the artichoke contain large amounts of sesquiterpenes, including cinnaratiol and cynararopicrin, which are believed to have a very intense bitter taste.

Artichoke seeds, which consist of 24% lipid, 21% protein and 17% fiber per mass of dry matter, are also valuable sources of various compounds. The seeds also contain large amounts of sterols, including β-sitosterol, campestrol, and 5-stigmasterol. In addition, artichoke seed oil is a valuable source of linoleic acid and oleic acid [5,14].

## 3. Biological Activity of Different Parts of *C. scolymus*

### 3.1. Antioxidant Activity

Artichoke and its various preparations, including the extracts from certain parts, exhibit antioxidant activity. For example, the luteolin-rich extract from raw artichoke flowers and leaves reduces the level of oxidative stress mediated by Cu^2+^ in vitro. Artichoke extract retarded the oxidation of low-density lipoprotein (LDL) in a dose-dependent manner, as indicated by the prolongation of the lag phase before conjugated diene formation, a decrease in the rate of propagation and the sparing of endogenous LDL alpha-tocopherol during oxidation. In addition, luteolin (1 µM) demonstrated a similar efficacy to artichoke extract (20 µg/mL) in inhibiting lipid peroxidation; it was also more effective than luteolin-7-*O*-glucoside at reducing LDL oxidation in a dose-dependent manner [17].

Zapolska-Downar et al. [18] also found artichoke to have antioxidative potential in an in vitro study of the influence of its aqueous and ethanolic extracts on intracellular oxidative stress and oxidative modification of LDL in endothelial cells and monocytes. The stress was stimulated by the application of tumor necrosis factor-α (TNFα) and lipopolysaccharide (LPS), which act as inflammatory mediators. Oxidative stress, associated with intracellular production of reactive oxygen species (ROS), was determined by measuring the oxidation of 2′, 7′-dichlorofluorescin (DCFH) to 2′, 7′-dichlorofluorescein (DCF). Both tested extracts inhibited basal and stimulated ROS production in endothelial cells and monocytes in a dose- and time-dependent manner. For example, in endothelial cells, the ethanolic extract (50 μg/mL) reduced ox-LDL-induced intracellular ROS production by 60% and the aqueous extract (50 μg/mL) by 43%. The ethanolic extract (50 μg/mL) also reduced ox-LDL-induced intracellular ROS production in monocytes by 76%.

Other in vitro studies by Vamanu and Vamanu [19] found the ethanolic extract (10 mg/mL) has antioxidant activity, as indicated by the 2,2-diphenyl-1-picrylhydrazyl (DPPH) assay. The extract also inhibited low-density lipoprotein oxidation and showed significant inhibitory activity against the tested strains of *Listeria innocua* CMGB 218, *Bacillus cereus* CMGB 215, with an MIC of 5 mg/mL.

Lim [14] suggests that the antioxidant activity of artichoke extracts may depend on the presence of phenolic compounds, mainly flavonoids, including luteolin and its derivatives. Indeed, Adzet et al. [20] report that a few phenolic compounds from *C. scolymus*, *viz.* chlorogenic acid, cynarin, isocholorogenic acid, quinic acid and caffeic acid, exhibit antioxidative properties, which were noted in isolated rat hepatocytes treated with carbon tetrachloride (CCl_4_).

Gebhardt and Fausel [21] report that aqueous artichoke extract decreases lipid peroxidation, measured as the production of MDA, in cultures of rat primary hepatocytes treated with *tert*-butyl hydroperoxide (t-BHP). Moreover, the tested extract prevented the corresponding loss of intracellular glutathione stimulated by t-BHP, which in turn induces lipid peroxidation. Similarly, in vitro studies on normal cell lines exposed to inducers of oxidative stress, including hydrogen peroxide (H_2_O_2_), also indicated that artichoke extract or its pure chemical compounds can prevent oxidative stress [22,23,24,25,26,27].

Llorach et al. [28] studied the antioxidant potential of commercial chicken soup supplemented with phenolic compound extracts (100 mg of phenolic compounds/g of dry extract) from various vegetables, including artichoke, lettuce and cauliflower. The cauliflower and lettuce extracts were composed of both caffeic acid derivatives and flavonols, while the artichoke extract contained caffeic acid derivatives and had the highest concentrations of phenolic compounds (100 mg of phenolic compounds/g of dry extract). In all cases, the extract was added to the soup to a maximum amount of 10 mg of extract per mL of soup. It was found that the addition of artichoke extract substantially increased the antioxidant capacity; the capacity was evaluated by FRAP assay, i.e., the ability to reduce the 2,4,6-tripyridyl-S-tri-azine (TPT2)-Fe(III) complex to TPT2-Fe(II), and ABTS^+^ assay, i.e., free radical scavenging activity, using 2,2′-azionobis (3-ethylbenzothiazoline-6-sulphonic acid).

A recent report by Vacca et al. [29] found that gluten-free bread enriched with artichoke leaf extract has both antioxidant and anti-inflammatory activity in vitro. The extract accounted for 5% of titratable chlorogenic acid.

Shallan et al. [30] studied the antioxidant potential of artichoke receptacles and bract extracts. The ethanolic extract of the bracts exhibited a higher total phenolic compound content, i.e., 56.3 µg gallic acid equivalent (GAE)/mg of extract, with catechin as the main component (16.04 mg/g of extract) compared to the extract from the receptacles. Moreover, the bract extract demonstrated stronger antioxidant potential than the receptacle extract: bract extract—6.42 µg/mL, receptacle extract—28.2 µg/mL (2,2-diphenyl-1-picrylhydrazyl assay); bract extract—32.7 µg/mL, receptacle extract—39.24 µg/mL (ABTS^+^ assay); bract extract—209.1 µmol/mL, receptacle extract—493.9 µmol/mL (FRAP assay).

Artichoke extracts have also been found to improve the oxidant-antioxidant balance in several models of healthy animals and those with various diseases. Their benefits have been noted in various organs, including the liver and kidney. Consumption of the edible portion of artichoke, representing about 14% of the calorie diet, resulted in a more favorable antioxidant status [31,32]. Kaymaz et al. [33] also demonstrated that aqueous artichoke leaf extract significantly decreases lipid peroxidation and increases the activity of various antioxidant enzymes, including catalase and superoxide dismutase, in rat hepatocytes. In addition, supplementation with artichoke leaves and artichoke leaf extract in rats decreased the level of oxidative stress following stimulation by CCl_4_ [34,35,36,37,38,39,40].

Moreover, artichoke extract has been found to have antioxidant potential in diabetic rats [41,42,43] and hypercholesterolemic rats [44,45,46,47,48]. For example, Magielse et al. [42] report that artichoke leaf extract (200 mg/kg) significantly decreased the level of various markers of oxidative stress, including the level of MDA in erythrocytes isolated from diabetic rats. Moreover, 60-day oral administration of artichoke leaf extract at two doses (200 and 400 mg/kg) significantly decreased oxidative stress in Wistar rats with cardiac damage and obesity induced by a high-fat diet [49]. In addition, treatment with ethanolic artichoke leaf extract (200 or 400 mg/kg/day for 60 days) also displayed antioxidant potential in the kidneys of Wistar rats fed a high-fat diet [50].

Recently, Florek et al. [51] found commercially available artichoke leaf powder extract, with a minimum caffeoylquinic acid content of 2.5% (Marlin Bauer group, Germany), to have antioxidant activity in the plasma and liver homogenate of rats with CCl_4_-induced liver damage. The antioxidant potential was estimated using various assays including TBARS, Trolox equivalent antioxidant capacity (TEAC) and glutathione peroxidase (GPx). For example, the authors observed that the concentration of TEAC in the liver homogenate was about 1 µmol/g of homogenate. The antioxidant property of artichoke has also been examined by a systematic review and meta-analysis of animal studies [10]. The studies indicate that artichoke extract supplementation increases liver glutathione levels and the activity of the antioxidative enzymes GPx, catalase (CAT), and superoxide dismutase (SOD) and decreases lipid peroxidation in plasma and liver.

While the antioxidant properties of artichoke have been confirmed in various in vitro and animal models, there have been very few clinical trials on the subject. For example, Rezazadeh et al. [52,53,54] found artichoke leaf extract supplementation (1800 mg/day for 12 weeks) to have antioxidant potential in 80 women with metabolic syndrome. This extract significantly decreased the ox-LDL level.

Skapanska-Stejnborn et al. [55] also report that supplementation with artichoke leaf extract (1200 mg/day for five weeks) demonstrated antioxidant potential in a group of rowers. Supplementation resulted in a significant increase in total antioxidant capacity; however, it also reduced glutathione concentration and did not change lipid peroxidation measured by TBARS. More details from various studies about the antioxidant potential of artichoke heads and leaves are demonstrated in Table 1.

Lattanzio et al. [3] studied the antioxidant activity of methanolic extracts of artichoke by-products, *viz.* the external bracts of artichoke heads, leaves and offshoots. These were found to be rich in phenolic compounds, including chlorogenic acid and 3,4-*O*-dicaffeoylquinic acid. They were also found to contain various phenolic compounds, including flavonoids, such as apigenin and luteolin glycosides, and tannins, such as hydrolysable and condensed tannins. The metmyoglobin assay and β-carotene/linoleate assay found the extracts, especially those from the artichoke heads, to demonstrate good antioxidant properties against hydroxyl and peroxyl radicals. Moreover, their results suggest that the artichoke offshoots may also be a potential source of natural antioxidants.

### 3.2. Effect on the Liver and Other Elements of Digestive System

Artichoke primarily owes its hepatoprotective effect to its antioxidant constituents, particularly its phenolic compounds [14,21,33,39,44,51,56,57,61,63,69,70,71,86,87,88]. For example, Colak et al. [39] report that treatment with artichoke leaf extract (1.5 g/kg/day for two weeks) supports antioxidant systems and reduces lipid peroxidation following CCl_4_-induced oxidative stress and hepatic injury. In addition, the extract supports the regulatory mechanism pathway, allowing DNA repair. Al-Ahdab [35] noted that both aqueous artichoke leaf and artichoke pulp extract have protective effects against CCl_4_-induced acute hepatotoxicity in rats. Hepatoprotective effects were noted at both tested doses (200 and 400 mg/kg).

Exposure to Cd can have toxic effects on the blood. El-Boshy et al. [40] found artichoke leaf extract (300 mg/kg bw) to protect against cadmium toxicity-stimulated hepatorenal damage and oxidative stress in rats and to reduce hematological disturbances induced by cadmium. Kaymaz et al. [33] also found aqueous artichoke leaf extract (1.5 g/kg orally for two weeks) to protect against hepatic damage stimulated by α-amanitine in rats (*n* = 28).

In addition, treatment with artichoke leaf powder capsules (100 mg/kg bw/day; 42 days) yielded a significant reduction in both non-enzymatic and enzymatic antioxidant potential and prevented histopathological abnormalities in liver tissues in male albino rats. In this experiment, hepatotoxicity was induced by aflatoxin (72 µg/kg bw) [69].

Sumer et al. [89] compared the hepatoprotective and nephroprotective action of various parts of artichoke, including receptacles, outer bracts, inner bracts, stems, and leaves, against paracetamol in rats. They observed that the stem and receptacle extracts of artichoke demonstrated higher protective effects than the leaf and bract extracts.

Recently, Doostkam et al. [68] studied the therapeutic action of milk thistle (Livergol^®^, Goldaru, Isfahan, Iran) and artichoke (Atheromod-B^®^) extracts on non-alcoholic fatty liver disease in type 2 diabetic rats. They found that the milk thistle extract reduced serum levels of total cholesterol, triglyceride, LDL and the serum activity of aspartate aminotransferase; the artichoke extract only decreased triglyceride levels. In addition, various systematic reviews and meta-analyses indicate that artichoke supplementation decreases aspartate aminotransferase and alanine aminotransferase in patients with non-alcoholic fatty liver diseases and obese or overweight subjects [90,91,92,93,94,95,96]. It has also been found that artichoke extracts improve non-alcoholic fatty liver disease and alcoholic liver disease in rodents [65,97,98]. For example, Tang et al. [97] report that ethanolic extract of artichoke (0.4–1.6 g/kg bw/day, for 10 days) also has a hepatoprotective effect, which was attributed to its ability to attenuate oxidative stress and suppress the Toll-like receptor 4 (TLR4)/nuclear factor-κB (NFκB) inflammatory pathway.

In addition, supplementation with standardized artichoke leaves and ginger root extracts, combined with simethicone, decreased digestive discomfort and altered gastric mobility in endurance athletes (*n* = 50). The subjects were treated for four weeks with two capsules containing 320 mg of artichoke and ginger extracts (Prodigest^®^, Technologiepark-Zwijnaarde, Gent, Belgia) plus 40 mg of simethicone [99]. Other studies found artichoke leaf extract to have beneficial effects on the gastrointestinal system in individuals with functional and mild dyspepsia and irritable bowel syndrome [76,80,100,101]. For example, Sabater et al. [101] found artichoke pectin and modified fractions (galactose- and arabinose-free) used at two doses (40 and 80 mg/kg) to have anti-inflammatory potential in mice with colitis. In addition, artichoke head extract was observed to demonstrate antiulcerogenic properties in rats [82].

### 3.3. Cardioprotective Action

Various animal and human models indicate that artichoke extracts and products, including its juices, also exert cardioprotective potential [102,103]. For example, Roghani-Dehkordi and Kamkhah [85] report that artichoke leaf juice reduces blood pressure in subjects with mild hypertension and Wang et al. [84] found artichoke bud extract to have beneficial effects in a rat model of hypertension. A recent meta-analysis of randomized controlled trials also found artichoke consumption to have anti-hypertensive properties in adults [92,93,94,95].

Artichoke may exert its cardioprotective properties via various mechanisms, including controlling obesity, oxidative stress, inflammation and lipid profile [102,103].

Artichoke extracts have also been found to have beneficial effects on dyslipidemia, one of the main risk factors for cardiovascular diseases (CVDs). In a study of the potential impact of artichoke consumption (leaf and heart extracts) on lipid profiles in humans, Santos et al. [104] observed that artichoke leaf extract decreased LDL concentration, total cholesterol, and triglyceride levels. It is believed that luteolin and chlorogenic acid, two components of this extract, play a key role in cardioprotection, while the beneficial effect of cooked artichoke hearts has been attributed mainly to its fibers, especially inulin. Shimoda et al. [105] found that sesquiterpenes also exert similar effects. For example, in the hepatocyte nucleus, luteolin reduces hepatocyte nuclear factor 4α (HNF4α) expression, consequently decreasing cholesterol synthesis due to inhibition of sterol-regulatory element-binding protein (SREBP) and 3-hydroxy-3-methylglutatryl-CoA reductase. In addition, inulin can modulate the lipid profile through the enterohepatic cycle [104]. More details about the effect of artichoke on lipid profiles and possible mechanisms of action have been described by Santos et al. [104].

A recent study by Cicero et al. [106] found patients with polygenic hypercholesterolemia (*n* = 60) to demonstrate significant improvements in several cardiovascular risk factors, including lipid profile, following treatment with artichoke extract.

A systematic review and meta-analysis by Sahebkar et al. [102] found artichoke extract supplementation to reduce total cholesterol, LDL and triglyceride levels, while another systematic review and meta-analysis by Majnooni et al. [91] confirmed that artichoke supplementation improves the lipid profile in patients with non-alcoholic fatty liver disease. Other studies have also demonstrated that artichoke supplementation appears to have a beneficial effect on lipid profiles in other models, including patients with hypercholesterolemia [78,79,81].

Arnaboldi et al. [103] report that supplementation with artichoke and bergamot extracts, either alone or in combination, has therapeutic potential in the treatment of mild to moderate dyslipidemia in patients with metabolic syndrome, hepatic steatosis or intolerance to statins or other drugs. In addition, Frigerio et al. [58] found artichoke leaf extract to have beneficial effects on cholesterol homeostasis in vitro.

Data also suggests that artichoke may have anti-obesity potential in rodents and humans [49,66,73,75,77]. This potential has been noted for artichoke leaf ethanol extract (160 mg/kg/day, for four weeks) in mice with diet-induced obesity [73], while artichoke consumption has been shown to have various effects, including inhibiting the action of digestive enzymes (lipase, α-glucosidase, and α-amylase), exerting a prebiotic effect, and enhancing lipolysis and lipid metabolism [107]. Inhibition of the secretion of inflammatory adipokines is also associated with the anti-obesity effect [108].

### 3.4. Other Biological Properties

Some papers indicate that various parts of artichoke also have neuroprotective properties [72,74,109].

A recent study by Ibrahim et al. [72] examined the neuroprotective effect of artichoke leaf extract (100 mg/kg bw, 42 days) against aflatoxin B_1_ (72 µg/kg bw) in male rats. The neurotoxicity of aflatoxin B_1_ was determined by *inter alia* biomarkers of oxidative stress, an increase in lipid profile, and an augmentation of glucose and insulin in plasma. The artichoke leaf extract was found to significantly reduce the adverse effects caused by aflatoxin B_1_. In addition, El-Nashar et al. [109] also report artichoke bract extract to have neuroprotective effects in an Alzheimer’s disease mouse model, and Cicek et al. [74] found artichoke left extract (0.8 and 1.6 g/kg; 14 days) to protect against diethylnitrosamine-induced (100 mg/kg) brain toxicity thanks to its antioxidant and antiapoptotic properties.

A meta-analysis of randomized clinical trials by Jalili et al. [110] found that supplementation with artichoke and artichoke products significantly reduces fasting blood sugar, although no other glycemic indexes (for example, fasting insulin) changed after administration. Fantini et al. [83] also demonstrated that artichoke head and leaf extracts have hypoglycemic properties in various in vivo models, including obese rats, diabetic rats, and patients with metabolic syndrome.

Some papers indicate that various other parts of artichoke, *viz.* the leaf, bracts, stem, and receptacles, also offer therapeutic potential in in vitro, in vivo or ex vivo models. They appear to demonstrate antifungal [30,111], antimicrobial [30], renoprotective [38,40,89], and anti-cancer potential [30,59,60,62,112,113], as well as an anti-arthritic effect [114]. For example, recently, Mathew et al. [59] observed that the extract from artichoke leaf (50 µg/mL, incubation time—72 h) inhibits the growth of the melanoma cell line HTB-72 by inhibiting proliferation and promoting apoptosis. Other studies demonstrated the anti-cancer activity of the extract from artichoke leaf in colon cancer cells [60], and this activity was also demonstrated for the extract from artichoke head in breast cancer cells in vitro [60].

**Figure 2:**
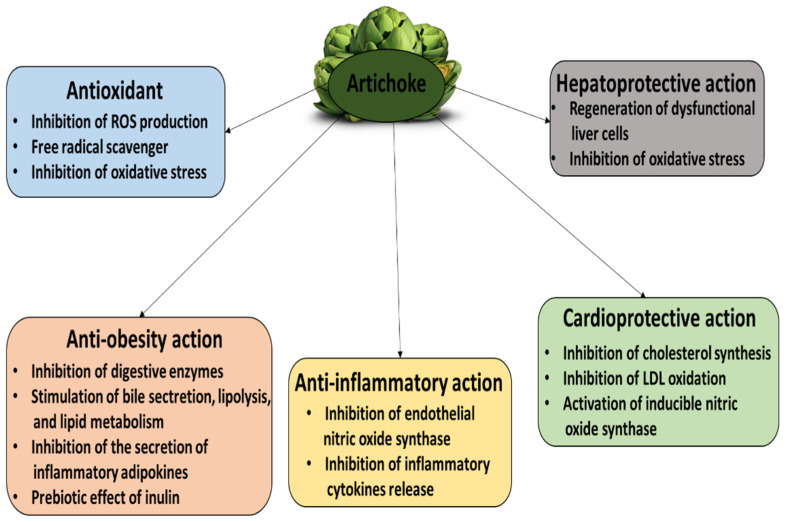
The biological properties of artichoke and its potential mechanisms of action.

More details about the various biological properties of artichoke parts based on in vivo and in vitro models are shown in Table 1. Figure 2 shows not only the biological properties of artichoke but also its potential mechanism of action.

## 4. The Safety of Artichoke and the Metabolism of Its Phenolic Compounds

In many countries, artichoke extracts, both dried and in solution, and the dried edible parts, including artichoke hearts, are currently commercialized as dietary supplements; these are typically sold as coated tablets or capsules intended for the treatment of liver diseases (Table 2). Some examples include “Artichoke” Herb Pharm (Williams, OR, USA), “Standardized Extract Artichoke” 500 mg, Jarrow Formulas (Santa Fe Springs, CA, USA), “Artichoke” (artichoke leaf extract, 250 mg), Paradise (Hayward, CA, USA), “Artichoke Hearts” 4.4 oz., “Artichoke Extract” (artichoke aerial parts extract, standardized to 5% cynarins) Source Naturals (San Jose, CA, USA), “Artichoke Extract”, 450 mg (min. 5% caffeoylquinic acid as cynarin), Now (Bloomingdale, IL, USA), “Cynara”, 200 mg, Vesta Phrmaceuticals (Indianapolis, IN, USA), “CINARAN^®^” (artichoke flowering head extract containing 13–18% of caffeolquinic acids), Indena S.p.A (Milan, Italy), “Artitoche” (artichoke leaf extract containing 0.3% flavonoids expressed as luteolin-7-*O*-glucoside and 2.5% caffeoylquinic acid expressed as cholorogenic acid), Indena S.p.A (Milan, Italy), [3,16].

The European Medicines Agency [115] recommends a daily dose of dried artichoke extract of 600–1320 mg; this amount has not demonstrated any side effects or signs of toxicity in rats, rabbits or mice. Englisch et al. [116] observed that the global tolerability of artichoke extract is excellent (95.7%). Data regarding the value of dietary supplements based on artichoke and their toxicity is given in more detail in Table 2.

After oral consumption of the cooked edible heads of artichoke, caffeolylquinic acid and chlorogenic acid are transformed into ferulic acid and caffeic acid conjugates by esterase throughout the small intestine and are later detected in human plasma. In addition, chlorogenic acid is hydrolyzed into aromatic acid metabolites, especially benzoic acid and coumaric acid, by colon enzymes. Interestingly, it has been found that the colon microflora plays an important role in releasing caffeic acid and its subsequent metabolism to dihydroferulic acid and dihydrocaffeic acid [107,117].

## 5. Conclusions

This review paper describes the current understanding of the versatility of different parts of globe artichoke (*Cynara scolymus* L.), its processing byproducts, and its use in dietary supplements. The most commonly exploited beneficial properties of artichoke and its products, especially dietary supplements, are arguably their antioxidant activity, cardioprotective action, and effect on the liver and the digestive system in general. In addition, artichoke consumption has also been associated with a range of health benefits, including prebiotic and probiotic potential (Table 1). However, only a few clinical trials have demonstrated any beneficial effect in humans, and the mechanisms by which artichoke consumption influences human health remain unclear. Nevertheless, it appears that the primary bioactive compounds involved in the pro-health potential of artichoke, its by-products and dietary supplements are phenolic compounds; even so, more studies are needed to clarify their precise mechanism of action.

## Figures and Tables

**Table 1:** Biological properties of various artichoke parts from different in vivo and in vitro models.

Artichoke Preparations	Dosage and Duration	Models	Biological Activity	References
In vitro model				
Leaf extract	50 µg/mL; 2 h	Human monocyte leukemia cell line exposed to LPS	Antioxidant activity	[27]
Leaf extract	1–100 µg/mL; 2 h	Human liver cells HepG2-ethanol-induced cell toxicity	Antioxidant activity; hepatoprotective activity	[56]
Leaf extract	25–100 µg/mL; 24 h	Monocytes and endothelial cells exposed to ox-LDL, LPS and TNFα	Antioxidant activity	[18]
Leaf extract	100 µg/mL and 1 ng/mL	Human leukocytes exposed to N-formyl-methionyl-leucyl-phenylalanine, phorbol myristate acetate, and H_2_O_2_	Antioxidant activity	[22]
Leaf extract	1–20 µg/mL	LDL	Antioxidant activity	[17]
Leaf extract	0.001–1 mg/mL; 40 min	Culture rat hepatocytes exposed to t-BHP	Antioxidant activity; hepatoprotective activity	[21]
Leaf extract	0.005 and 0.5 mg/mL; 40 min	Culture rat hepatocytes exposed to H_2_O_2_	Antioxidant activity; hepatoprotective activity	[57]
Leaf extract	50–250 µg/mL; 48 h	Human hepatic cell line (HepG2)	Anticholesterolemic activity	[58]
Leaf extract	50 µg/mL; 72 h	Melanoma cell line HTB-72	Anti-cancer activity	[59]
Leaf extract	1–4 µg/mL; 4 h	Human HT-29 and RKO colon cancer cells	Anti-cancer activity	[60]
Head extract	0.18–1.44 µg/mL; 1–4 h	Human LDL	Antioxidant activity	[26]
Head extract	0.75–24 µg/mL; 30 min	Human intestinal cell line (HT-29) exposed to H_2_O_2_	Antioxidant activity	[25]
Head extract	400–1200 µM; 24 h	Human hepatoma HepG2 cells and cultured rat hepatocytes exposed to glucose oxidase	Antioxidant activity; hepatoprotective activity	[61]
Head extract	12.5–50 µM; 24 h	Human breast cell lines (MCF7 and MDA-MB231)	Anti-cancer activity	[62]
Head extract	5 µg/L; 24 or 48 h	Colonic microbiota	Prebiotic effect	[12]
In vivo model				
Leaf extract	200 and 400 mg/kg; 4 weeks	Diabetic rats	Antioxidant activity; hypoglycaemic action	[43]
Leaf extract	200 and 400 mg/kg; 60 days	Rats with obesity	Antioxidant activity; renoprotective activity	[50]
Leaf extract	300 mg/kg; 4 weeks	Rats treated with Cd	Antioxidant activity; renoprotective; hepatoprotective activity; reducing hematological disturbances	[40]
Leaf extract	1500 mg/kg; 2 weeks	Rats with α-amanitine induced hepatotoxicity	Antioxidant activity; hepatoprotective activity	[33]
Leaf extract	1500 mg/kg; 2 weeks	Rats with CCl_4_ induced hepatotoxicity	Antioxidant activity; hepatoprotective activity	[39]
Leaf extract	125–500 mg/kg; 4 weeks	Rats with ethylene glycol-induced urolithiasis	Antioxidant activity	[37]
Leaf extract	200–600 mg/kg; 10 days	Rats with gentamicin-induced nephrotoxicity	Antioxidant activity; renoprotective activity	[38]
Leaf extract	150–600 mg/kg; 30 days	Hypercholesterolemic rats	Antioxidant activity	[48]
Leaf extract	1500 and 3000 mg/kg; 42 days	Hypercholesterolemic rats	Antioxidant activity	[47]
Leaf extract	1500 mg/kg; 2 weeks	Rats with paracetamol-induced liver injury	Antioxidant activity; hepatoprotective activity	[63]
Leaf extract	200 mg/kg; 30 days	Rats with 5-flurouracil induced cardiotoxicity	Antioxidant activity	[36]
Leaf extract	20% and 40% of diets; 4 weeks	Rats treated with CCl_4_	Antioxidant activity	[34]
Leaf extract	200 and 1000 mg/kg; 3 weeks	Diabetic rats	Antioxidant activity	[42]
Leaf extract	20–80 mg/kg; 36 days	Aging rats	Antioxidant activity	[64]
Leaf extract	200 and 400 mg/kg; 21 days	Diabetes rats	Antioxidant activity	[41]
Leaf extract	0.4, 0.8, and 1.6 g/kg bw; 8 weeks	Rats treated with a high-fat diet	Antioxidant activity; hepatoprotective activity; anti-inflammatory effect; anti-insulin resistance effect	[65]
Leaf extract-rich in luteolin	0.005%; 16 weeks	Mice with high-fat diet-induced obesity	Anti-obesity effect	[66]
Leaf extract	1500 mg/kg; 2 weeks	Hypercholesterolemic rats	Antioxidant activity; hepatoprotective activity	[44]
Leaf extract	1500 mg/kg; 2 weeks	Hypercholesterolemic rats	Antioxidant activity	[45]
Leaf extract	1500 mg/kg; 2 weeks	Rats treated with CCl_4_	Antioxidant activity	[67]
Leaf extract	200 and 400 mg/kg; 6 weeks	Rats treated with CCl_4_	Antioxidant activity; hepatoprotective activity	[35]
Leaf extract	200 and 400 mg/kg; 60 days	Wistar rats with a high-fat diet	Antioxidant activity; cardioprotective action	[49]
Leaf extract	0.5, 1, and 1.5 g/kg bw/day; 2 weeks	Rats treated with CCl_4_	Antioxidant activity	[51]
Leaf extract (Atheromod-B^®^, Barij Essence Pharmaceutical Co., Tehran, Iran)	60 mg/kg/day; 8 weeks	Type 2 diabetic rats	Antioxidant activity; cardioprotective action; hepatoprotective effect (no date)	[68]
Leaf extract	100 mg/kg/day; 42 days	Male albino rats treated with aflatoxin B_1_	Antioxidant activity; hepatoprotective effect	[69]
Leaf extract	1500 mg/kg/day; 15 days	Rats	Antioxidant activity; hepatoprotective effect	[70]
Leaf extract	30 mg/kg	Rats	Antioxidant activity; hepatoprotective effect	[71]
Leaf extract	100 mg/kg; 42 days	Rats treated with aflatoxin B_1_	Neuroprotectory effect	[72]
Leaf extract	1600 mg/kg/day; 4 weeks	Mice with obesity	Antioxidant activity; hepatoprotective effect; anti-obesity potential	[73]
Leaf extract	0.8 and 1.6 g/kg/day; 2 weeks	Mice treated with diethylnitrosamine	Neuroprotectory effect	[74]
Leaf extract	500 mg twice daily, 8 weeks	Hypertensive patients	Improving BMI and systolic blood pressure	[75]
Leaf extract	1800 mg/day; 12 weeks	Patients with metabolic syndrome	Antioxidant activity	[52]
Leaf extract	1200 mg/day; 5 weeks	Members of a rowing team	Antioxidant activity	[55]
Leaf extract (HeparSL^®^ forte, MCM Klosterfrau Vert. Gmbh, Berlin, Germany)	320 mg/day; 6 weeks	Patients with functional dyspepsia	Alleviating symptoms and improving the disease-specific quality of life	[76]
Leaf extract	1800 mg/day; 12 weeks	Patients with metabolic syndrome	Hypoglycemic action	[77]
Leaf extract	250 mg/day; 8 weeks	Overweight subjects with mild hypercholesterolemia	Cardioprotective action	[78]
Leaf extract	250 mg, twice daily; 60 days	Adults with low HDL-cholesterol and mild hypercholesterolemia	Cardioprotective action	[79]
Leaf extract	320 or 640 mg/day; 2 months	Otherwise healthy subjects suffering from dyspepsia	Alleviating symptoms and improving the disease-specific quality of life	[80]
Leaf extract	1280 mg/day; 3 months	Healthy adults with mild to moderate hypercholesterolemia	Cardioprotective action	[81]
Head extract	1500 and 3000 mg/kg; 42 days	Hypercholesterolemic rats	Antioxidant activity	[47]
Head extract	14% of diets; 3 weeks	Normal rats	Antioxidant activity	[32]
Head extract	14% of diets; 3 weeks	Normal rats	Antioxidant activity	[31]
Head extract	200 and 400 mg/kg; 2 weeks	Rats	Antiulcerogenic activity	[82]
Head extract	500–1500 mg/kg	Normal and obese rats	Hypoglycemic action	[83]
Pulp (hearts and heads) extract	200 and 400 mg/kg; 6 weeks	Rats treated with CCl_4_	Antioxidant activity	[35]
Artichoke pellets (made by mixing dried and crushed artichoke)	10% of diets; 45 days	Hyperlipidemic rats	Antioxidant activity	[46]
Bud extract	50 and 100 mg/kg/day; 4 weeks	Hypertensive rats	Anti-hypertension effect	[84]
Leaf juice	50 and 100 mg juice concentrate; 12 weeks	Patients with mild hypertension	Anti-hypertension effect	[85]

**Table 2:** Dietary supplements with artichoke used in selected countries, based on European Medicines Agency [115] and by Mahboubi [107].

Country	Dietary Supplement	Diseases	Advance Effect
Austria	1–2 coated tables or capsules (350 mg), three times daily	Digestive complaints, dyspepsia	No advance effect known
Belgium	Three coated tables or capsules (200 mg), two times daily	Digestive complaints, dyspepsia	No advance effect known
Bulgaria	1–2 coated tablets, three times daily, or 1–2 teaspoon of solution, three times daily	Dyspepsia, and enhancing the fatty acid metabolism	Hypersensibility, diarrhea, nausea, flatulence
France	1–2 coated tablets (200 mg), three times daily, or 1 ampule (10 mL), three times daily	Digestive and urinary complaints, dyspepsia	No advance effect known
Germany	One coated tablet (300 mg), 1–2 times daily	Digestive complaints, dyspepsia	Slight diarrhea, epigastric complaints
Poland	Two tablets, one a day (digestive disorders) or 2 tablets, three times daily (hyperlipidaemia)	Digestive complaints and hepatobiliary disturbances, mild to moderate hyperlipidemia	Mild gastro-intestinal disturbances
Spain	600–1500 mg a day–capsules	Dyspepsia	No advance effect known
United Kingdom	one capsule, twice daily	Digestive complaints	No advance effect known

## Abbreviations

ABTS^+^—2,2′-azionobis (3-ethylbenzothiazoline-6-sulphonic acid); bw—body weight; CVDs—cardiovascular diseases; DCFH—2′, 7′-dichlorofluorescin; DCF—2′, 7′-dichlorofluorescein; DPPH—2,2-diphenyl-1-picrylhydrazyl; FRAP—ferric reducing antioxidant power; LDL—low density lipoprotein; LPS—lipopolysaccharide; MDA—malondialdehyde; NF-κB—nuclear factor kappa B; oxLDL—oxidative modification of LDL; ROS—reactive oxygen species; t-BHP—*tert*-butyl hydroperoxide; TBARS—thiobarbituric acid reactive substances; TEAC—Trolox equivalent antioxidant capacity; TLR4—Toll-like receptor 4; TNF-α—tumor necrosis factor-α.
